# Burnout in medical residents: the combined influence of on-call burden, somatic symptomatology, insomnia severity, and physical activity

**DOI:** 10.3389/fspor.2026.1779677

**Published:** 2026-05-29

**Authors:** Hamdi Henchiri, Amr Chaabeni, Hela Znazen, Jaouher Hamaidi, Medina Srem-Sai, John Elvis Hagan, Chayma Harrathi, Vlad Adrian Geantă, Abdullah H. Alliheibi, Waleed Ghanim Thanoon, Anis Jellad, Fairouz Azaiez

**Affiliations:** 1 Higher Institute of Sport and Physical Education of Sfax, University of Sfax, Sfax, Tunisia; 2 Higher Institute of Sport and Physical Education of Gafsa, University of Gafsa, Gafsa, Tunisia; 3 Research Laboratory of Education, Motricité, Sport et Santé (LR19JS01), Sfax, Tunisia; 4 Occupational Medicine and Professional Pathologies Service, Gafsa Regional Hospital, Gafsa, Tunisia; 5 Research Laboratory of Technology and Medical Imaging (LR12ES06), Center for Musculoskeletal Biomechanics Research, Faculty of Medicine, University of Monastir, Monastir, Tunisia; 6 Department of Physical Medicine and Rehabilitation, Faculty of Medicine, University of Monastir, Monastir, Tunisia; 7 Department of Sport Health, College of Sport Sciences and Physical Activity, Princess Nourah Bint Abdulrahman University, Riyadh, Saudi Arabia; 8 Higher Institute of Sport and Physical Education of El Kef, University of Jendouba, Jendouba, Tunisia; 9 Department of Health, Physical Education, Recreation and Sports, University of Education, Winneba Ghana; 10 Department of Health, Physical Education and Recreation, University of Cape Coast, Cape Coast, Ghana; 11 Neurocognition and Action - Biomechanics Research Group, Faculty of Psychology and Sports Science, Bielefeld University, Bielefeld, Germany; 12 Department of Physical Education and Sport, Faculty of Physical Education and Sport, Aurel Vlaicu University of Arad, Arad, Romania; 13 Department of Physical Education, College of Sport Sciences and Physical Activity, King Saud University, Riyadh, Saudi Arabia; 14 College of Physical Education and Sport Science, University of Mosul, Mosul, Iraq

**Keywords:** burnout, insomnia, medical residents, physical activity, somatic symptoms, workload

## Abstract

**Introduction:**

Burnout has emerged as a critical threat to healthcare systems, particularly among medical residents who face intense workloads, recurrent night shifts, and emotionally demanding clinical environments. In Tunisia, evidence on the interplay between occupational, psychological, and lifestyle determinants of burnout remains limited. This study aimed to determine the prevalence of burnout among Tunisian medical residents and to identify key predictors, including on-call burden, somatic symptomatology, insomnia severity, substance use, and physical activity, using a multidimensional analytic approach.

**Methods:**

A multi-center cross-sectional study was conducted across six Tunisian university hospitals from March 2024 to January 2025. Medical residents aged 25–35 years completed validated instruments, including the Maslach Burnout Inventory–Human Services Survey (MBI-HSS), Insomnia Severity Index, Somatic Symptom Scale-8, and Ricci–Gagnon physical activity questionnaire. Sociodemographic, professional, and behavioral factors were also collected. Pearson correlation analyses and multivariate logistic regression were performed to identify factors associated with burnout.

**Results:**

Among 320 residents, burnout prevalence was 45.9%, with pathological mean scores across all MBI-HSS subscales. Emotional exhaustion strongly correlated with somatic symptom burden (*r* = 0.84), insomnia severity (*r* = 0.75), and inversely with physical activity (*r* = −0.82). Logistic regression revealed that the number of monthly on-call shifts was associated with burnout (OR = 2.72; *p* < 0.001). Intrinsic motivation for choosing medicine was associated with lower odds of burnout (OR = 0.13; *p* = 0.001). Higher somatic symptoms were associated with burnout risk (OR = 1.20; *p* = 0.013), whereas physical activity may reduce it (OR = 0.82; *p* = 0.001).

**Conclusions:**

Burnout is highly prevalent among Tunisian medical residents and is shaped by a combination of occupational strain, psychosomatic distress, and lifestyle behaviors. On-call overload, somatic symptoms, and poor sleep were associated with the increase of burnout risk, while regular physical activity and intrinsic career motivation may serve as protective factors. These findings support the implementation of targeted preventive strategies and early screening tools within residency training programs.

## 1 Introduction

Health care is a basic part of every society and has a big effect on both the economy and society. This is because the health of the population is closely linked to the growth of society (1, 2). Individuals who experience health problems are unable to work efficiently, leading to labor shortages, decreased productivity, and increased costs for employers, while also placing an additional burden on the state (3). Numerous studies have demonstrated a clear relationship between population health and workplace productivity (4, 5). Consequently, maintaining high-quality health care requires that health care workers themselves be physically, psychologically, and socially healthy. In this regard, the provision of appropriate working conditions is essential to ensure optimal quality of care (6–8).

Burnout syndrome is a work-related condition primarily associated with prolonged exposure to excessive emotional, personal, cognitive, and physical stress, and is characterized by a negative attitude toward work (9). The syndrome is classically defined by three core dimensions: emotional exhaustion, depersonalization, and a reduced sense of personal accomplishment (10).

Worldwide, studies have shown that burnout affects a substantial proportion of health care workers (11–14). Among them, medical residents appear to be particularly vulnerable due to the nature of their training and professional responsibilities (15, 16). Residents are routinely exposed to demanding working conditions, including long working hours, frequent night shifts, heavy patient workloads, and emotionally challenging clinical situations, all of which contribute to increased psychological strain (17, 18).

Recent studies conducted in different regions have reported a high prevalence of burnout among medical residents. In Saudi Arabia, for instance, 30.3% of residents reported high emotional exhaustion, 24.9% experienced high depersonalization, and 86.3% reported a reduced sense of personal accomplishment (19). Similarly, a study in Thailand found that 46.3% of residents met the criteria for burnout, with 57.1% suffering from emotional exhaustion and 36.1% from depersonalization (20). In Mauritania, another study revealed that 35% of residents had high emotional exhaustion, 36% reported depersonalization, and 73% experienced low personal accomplishment (21). Research conducted in Tunisia consistently confirms that burnout is a widespread and serious issue among medical residents, with a significant proportion experiencing severe emotional exhaustion, depersonalization, and reduced personal accomplishment (22–25).

Burnout syndrome has considerable consequences at both the individual and societal levels (9, 26). It may lead to emotional depletion, loss of energy, dehumanization, detachment from work, feelings of inadequacy, reduced productivity, and impaired coping abilities (13, 27), Moreover, burnout is associated with various mental and somatic complications, including depression, insomnia, cardiovascular disorders, and chronic pain syndromes (28–31). It may also contribute to substance use, such as tobacco, alcohol, or illicit drugs, as well as the development of addictive behaviors (32–34).

The World Health Organization defines physical activity as any bodily movement produced by skeletal muscles that requires energy expenditure (35). This includes physical activity undertaken during leisure time, transportation, work, or domestic activities. Physical activity is essential in both preventive and therapeutic approaches, impacting physical, psychological, cognitive, and social dimensions (36–38). Studies across different age groups and genders have demonstrated that regular physical activity promotes psychological and physical well-being and contributes to improved productivity at work and enhanced academic performance (39, 40). Despite this growing body of evidence, the role of physical activity in relation to mental health and quality of life remains a subject of ongoing debate.

Regarding burnout specifically, the literature presents mixed findings. Several studies have highlighted the protective role of physical exercise, demonstrating its effectiveness in reducing burnout symptoms and mitigating its core dimensions (41–43). Conversely, other studies have failed to establish a strong association between physical activity and burnout levels (42, 44). These discrepancies may be explained by differences in exercise type, intensity, frequency, and the professional contexts of the study populations.

The present study aimed to determine the prevalence of burnout among Tunisian medical residents and to identify its key determinants. More specifically, the study examined the influence of workload-related factors (particularly the number of on-call shifts), psychological and somatic symptoms (including insomnia severity and somatic complaints), lifestyle behaviors (physical activity, tobacco use, and alcohol consumption), and motivational orientation (intrinsic vs. extrinsic career choice) on the development of burnout. A further objective was to construct an exploratory logistic regression model to identify factors associated with increased burnout risk and to provide a preliminary framework for future predictive research.

## 2 Materials and methods

### 2.1 Study design

This study employed a multi-center cross-sectional design conducted between March 1, 2024, and January 2025. Medical residents from six Tunisian university hospitals were recruited, representing a range of specialties. The study was designed and reported in accordance with the STROBE guidelines.

### 2.2 Participants

Eligible participants were medical residents aged 25–35 years with at least six months of residency training. Residents were excluded if they had a diagnosed psychological disorder or a contraindication for exercise. Participants were recruited on a voluntary basis through institutional email invitations and direct contact during departmental meetings across the participating hospitals. A total of 340 residents were approached, of whom 320 agreed to participate and completed the survey, yielding a response rate of 94.1%. The presence of psychological disorders was assessed based on self-reported previous medical diagnosis. The study protocol was reviewed and approved by the Ethics Committee of the Faculty of Medicine of Monastir (approval number: IORG 0009738, N° 122/OMB 0990-0279). All participants provided written informed consent prior to their inclusion in the study.

### 2.3 Measures

Data were collected using a structured questionnaire that included sociodemographic, socio-professional, and general health-related variables. These comprised gender, age, place of residence, medical specialty, night-shift work, choice of specialty, marital status, and tobacco and alcohol use. For analytical purposes, medical specialties were categorized into two groups: acute/emergency-oriented specialties (including emergency medicine, anesthesiology and intensive care, trauma surgery, cardiology, neurosurgery, general surgery, and obstetrics) and non-emergency/chronic care specialties (such as endocrinology, dermatology, rheumatology, psychiatry, physical medicine and rehabilitation, occupational medicine, and geriatrics).

Burnout was assessed using the Arabic validated version of the Maslach Burnout Inventory–Human Services Survey (MBI-HSS), as previously validated among Tunisian medical residents. The scale comprises nine items for Emotional Exhaustion, five items for Depersonalization, and eight items for Personal Accomplishment. Burnout was defined as the simultaneous presence of high emotional exhaustion (EE ≥ 27), high depersonalization (DP ≥ 10), and low personal accomplishment (PA ≤ 33), in accordance with established cut-off values (10, 45). This composite definition has been widely used in previous research employing the MBI.

Insomnia symptoms were evaluated using the Insomnia Severity Index (ISI) (46), a 7-item self-report questionnaire designed to assess the nature, severity, and impact of insomnia. The ISI measures difficulties with sleep initiation, sleep maintenance, early morning awakenings, dissatisfaction with sleep, interference with daily functioning, noticeability of sleep problems, and associated distress. Total scores range from 0 to 28, with higher scores indicating greater insomnia severity.

Somatic symptom burden was assessed using the Somatic Symptom Scale–8 (SSS-8) (47), a brief 8-item self-report instrument that evaluates the severity of common somatic symptoms experienced over the past week. The scale covers gastrointestinal complaints, pain, fatigue, dizziness, and cardiopulmonary symptoms. Total scores range from 0 to 32, providing a rapid and reliable index of overall somatic symptom burden.

Physical activity levels were measured using the Ricci–Gagnon questionnaire (48), a validated self-report tool for assessing habitual physical activity. The questionnaire evaluates both occupational and leisure-time physical activity by quantifying the frequency, duration, and intensity of daily movements. Based on the total score, participants are classified into categories such as inactive, moderately active, or active, offering a concise and reliable estimate of overall physical activity.

Substance use was operationalized as tobacco and alcohol consumption, representing the most prevalent and reliably self-reported forms of substance-related behaviors in this population.

### 2.4 Sample size calculation

In the absence of robust national prevalence estimates at the time of study design, the sample size calculation was based on previously published data from comparable resident populations, a commonly accepted approach in epidemiological research.

Based on an expected burnout prevalence of 18.3% reported among Saudi resident physicians, the minimum required sample size was estimated at 317 participants. This calculation assumed a margin of error of ±4%, a 90% confidence level, and included an anticipated non-response rate of 20%. The sample size estimation was performed using the Scalex SP sample size calculator.

### 2.5 Statistical analysis

Statistical analyses were conducted using SPSS software, version 26 (IBM Corp., Armonk, NY, USA). Quantitative variables were expressed as means ± standard deviations (SD), while qualitative variables were presented as frequencies and percentages. Associations between burnout and categorical variables were assessed using the chi-square test, and comparisons of continuous variables were performed using Student's *t*-test. Variables with a *p*-value < 0.20 in univariate analyses were subsequently entered into a multivariate logistic regression model using stepwise backward elimination. The number of on-call shifts per month was entered into the regression model as a continuous variable. The linearity-in-the-logit assumption was not formally tested; therefore, the associated odds ratio should be interpreted with caution as reflecting the cumulative association across the observed range rather than a strict per-unit effect. Adjusted odds ratios (ORs) with corresponding 95% confidence intervals (CIs) were reported. Before multivariable regression analysis, multicollinearity among independent variables was assessed using variance inflation factor (VIF), tolerance values, and condition indices. Multicollinearity was considered problematic when VIF > 5 (or 10), tolerance < 0.20.

Statistical significance was defined as *p* < 0.05.

## 3 Results

### 3.1 Internal consistency of the maslach burnout inventory-HSS

The internal consistency of the Arabic version of the Maslach Burnout Inventory–Human Services Survey (MBI-HSS) was evaluated using Cronbach's alpha for each of the three subscales. The Emotional Exhaustion subscale demonstrated excellent internal reliability (α = 0.977) across nine items. The Depersonalization subscale also showed high internal consistency (α = 0.879; five items). Similarly, the Personal Accomplishment subscale exhibited strong reliability (α = 0.926; eight items). Overall, these findings indicate that the Arabic version of the MBI-HSS has robust psychometric properties in the studied population of Tunisian medical residents.

Although the Cronbach's alpha for the Emotional Exhaustion subscale was very high, this finding may also suggest a degree of item redundancy, which should be considered when interpreting internal consistency results.

### 3.2 Prevalence of burnout among tunisian medical residents

The overall prevalence of burnout among medical residents was 45.9%. It should be noted that the 61.3% figure reported in Table 1 refers to the proportion of participants with low personal accomplishment as an isolated dimension, whereas the composite burnout prevalence, defined as the simultaneous presence of high emotional exhaustion, high depersonalization, and low personal accomplishment was 45.9% (*n* = 147, out of 320 participants). This composite definition served as the binary outcome variable in the logistic regression and ROC analyses. The sociodemographic, occupational, and clinical characteristics of the study population are presented in Table 1. Participants had a mean age of 27.63 ± 2.04 years, with a predominance of females. Most residents were single, and nearly half reported tobacco use, while fewer than one third reported alcohol consumption. The mean number of on-call shifts per month was 6.6 ± 3.3, with an average of 0.6 ± 0.7 on-call weekends per month. The majority of participants reported dissatisfaction with their salary. Mean scores for somatic symptoms, insomnia severity, and physical activity are also detailed in Table 1.

**Table 1:** Socio-demographic, occupational, lifestyle, and clinical characteristics of Tunisian medical residents (*N* = 320).

Variable	Value
Burnout (*n*/%)	147/45.9
High Emotional Exhaustion (EE ≥ 27) (*n*/%)	189/59
High Depersonalization (DP ≥ 10) (*n*/%)	181/56.6
Low Personal Accomplishment (PA ≤ 33) (*n*/%)	196/61.3
Age (mean ± SD)	27.63 ± 2.04
Gender (*n*) (Male/Female)	130/190
Marital status (*n*/%)	
Single	269/84
Married	51/16
Smoking (*n*/%)	147/45.9
Alcohol (*n*/%)	94/29.4
Specialty (*n*)	
Emergency/Non-emergency	203/117
Medical/Surgical	184/136
Number of on-call per month (mean ± SD)	6.6 ± 3.3
Number of on-call weekends per month (mean ± SD)	0.6 ± 0.7
Salary satisfaction (*n*/%)	
Satisfied	50/15.6
Not satisfied	270/84.4
Somatic symptom scale (mean ± SD)	13.8 ± 5.8
Insomnia Severity Index (mean ± SD)	16.9 ± 5.4
Physical activity (mean ± SD)	20.8 ± 7.6
Ricci–Gagnon questionnaire (*n*/%)	
Active	166/51.9
Not active	154/48.1

Data are presented as mean ± standard deviation (SD) for continuous variables and as number (n) and percentage (%) for categorical variables. Burnout was assessed using the Maslach Burnout Inventory–Human Services Survey (MBI-HSS). Physical activity was evaluated using the Ricci–Gagnon questionnaire, with higher scores indicating greater levels of habitual physical activity.

### 3.3 Burnout dimensions according to gender

Mean scores for the three MBI-HSS subscales are shown in Table 2. In the overall sample, mean levels of Emotional Exhaustion and Depersonalization, as well as reduced Personal Accomplishment, were within the pathological range. Similar patterns were observed in both male and female residents.

**Table 2:** Maslach burnout inventory-human services survey (MBI-HSS) values among medical residents.

Variable	All participants (*N* = 320)	Males (*N* = 130)	Females (*N* = 190)	*p* value
Emotional Exhaustion	34.62 ± 15.42	34.08 ± 15.89	34.99 ± 15.11	0.681
Depersonalization	19.06 ± 8.16	18.99 ± 8.51	19.10 ± 7.92	0.914
Personal Accomplishment	20.98 ± 16.26	20.55 ± 16.27	21.28 ± 16.29	0.704

Data are presented as mean ± standard deviation (SD). Burnout dimensions were assessed using the Maslach Burnout Inventory–Human Services Survey (MBI-HSS). Higher scores on Emotional Exhaustion and Depersonalization indicate greater burnout severity, whereas lower scores on Personal Accomplishment reflect higher burnout. Pathological cut-off values were defined as Emotional Exhaustion ≥ 27, Depersonalization ≥ 10, and Personal Accomplishment ≤ 33.

These findings indicate that burnout severity was comparable between genders, suggesting that gender did not significantly influence burnout dimensions in this population.

### 3.4 Correlation analysis

Pearson correlation analyses between workload indicators, somatic symptoms, insomnia severity, physical activity, and the three dimensions of burnout are presented in Table 3.

**Table 3:** Pearson correlation matrix between workload indicators, clinical variables, and burnout dimensions.

Variable	(1)	(2)	(3)	(4)	(5)	(6)	(7)
On-call shifts per month (1)	1.00						
Somatic Symptom Scale (SSS) score (2)	.38**	1.00					
Insomnia Severity Index (ISI) score (3)	.33**	.78**	1.00				
Physical activity (4)	−.45**	−.75**	−.69**	1.00			
Burnout dimensions							
Emotional Exhaustion (5)	.43**	.84**	.75**	−.82**	1.00		
Depersonalization (6)	.37**	.02	−.04	.08	−.05	1.00	
Personal Accomplishment (7)	−.50**	−.79**	−.68**	.78**	−.87**	−.03	1.00

*N* = 320. Values represent Pearson correlation coefficients. Higher scores on Emotional Exhaustion and Depersonalization indicate greater burnout severity, whereas lower scores on Personal Accomplishment reflect higher burnout.

For clarity, the variables included in the correlation matrix are presented in the following order: (1) on-call shifts per month, (2) somatic symptoms, (3) insomnia severity, (4) physical activity, (5) emotional exhaustion, (6) depersonalization, and (7) personal accomplishment.

* *p* < 0.05.

** *p* < 0.01.

Emotional exhaustion showed strong positive correlations with the number of on-call shifts per month (*r* = 0.43, *p* < 0.01), somatic symptom burden (*r* = 0.84, *p* < 0.001), and insomnia severity (*r* = 0.75, *p* < 0.001). A strong negative correlation was observed between emotional exhaustion and physical activity (*r* = −0.82, *p* < 0.001).

Depersonalization was significantly correlated only with the number of on-call shifts per month (*r* = 0.37, *p* < 0.01) and showed no significant associations with somatic symptoms, insomnia, or physical activity. This suggests that depersonalization may be more closely related to workload factors than to psychological or somatic variables in this population.

Personal Accomplishment demonstrated strong negative correlations with the number of on-call shifts, somatic symptom burden, and insomnia severity, while being positively correlated with physical activity levels.

Multicollinearity diagnostics showed acceptable levels of collinearity among predictors. Tolerance values ranged from 0.300 to 0.620, while VIF values ranged from 1.612 to 3.338, all below commonly accepted thresholds for problematic multicollinearity (VIF < 5 and tolerance > 0.20).

These findings suggest that higher physical activity levels were associated with better perceived professional efficacy, whereas greater workload and psychological distress were associated with lower personal accomplishment.

### 3.5 Determinants of burnout: multivariate logistic regression

Variables retained in the final multivariate logistic regression model are summarized in Table 4. The fitted model can be expressed as:

**Table 4:** List of variables derived from the backward method.

Variable	B	S.E	Wald	df	Sig.	Exp(B)	95% C.I.for EXP(B)	
							Lower	Upper
On-call shifts per month	1.002	.140	51.241	1	.000	2.722	2.070	3.581
Field of study choice	−2.050	.621	10.902	1	.001	.129	.038	.435
Tobacco use	.992	.548	3.282	1	.070	2.697	.922	7.890
Alcohol consumption	.951	.564	2.848	1	.091	2.589	.858	7.814
Somatic Symptom	.182	.073	6.142	1	.013	1.199	1.039	1.385
Physical Activity	−.198	.058	11.579	1	.001	.820	.732	.919
Constant	−4.964	2.211	5.040	1	.025	.007		

S.E, standard error of the regression coefficient; df, degrees of freedom; Sig., significance (*p*-value); Exp(B), exponentiated coefficient (odds ratio); C.I. for Exp(B), confidence interval for the odds ratio.

Logit (Burnout) = −4.964 + 1.002 × on-call shifts per month − 2.050 × field of study choice + 0.992 × tobacco use + 0.951 × alcohol consumption + 0.182 × somatic symptoms − 0.198 × physical activity.

A higher number of on-call shifts per month was associated with increased odds of burnout (OR = 2.72; 95% CI: 2.070–3.581; *p* < 0.001), indicating a positive association between on-call workload and burnout within this exploratory association model.

Field of study choice was also associated with burnout. Residents who reported choosing medicine based on personal interest had lower odds of burnout compared with those influenced by external pressures, particularly family expectations (*B* = −2.050; *p* = 0.001; OR = 0.129; 95% CI: 0.038–0.435), suggesting an association between intrinsic motivational orientation and reduced burnout risk within this exploratory risk-stratification model.

Somatic symptom burden was associated with higher odds of burnout (*B* = 0.182; *p* = 0.013; OR = 1.199; 95% CI: 1.039–1.385), indicating a positive association between somatic symptoms and burnout. In contrast, physical activity was associated with lower odds of burnout (*B* = −0.198; *p* = 0.001; OR = 0.820; 95% CI: 0.732–0.919), suggesting a potential protective association within this exploratory framework.

Tobacco and alcohol use were associated with higher odds of burnout; however, these associations did not reach statistical significance in the adjusted model (*p* = 0.070 and *p* = 0.091, respectively), indicating no clear independent association after adjustment for other variables in the model, suggesting potential confounding or collinearity with the retained predictors.

Although insomnia severity showed strong bivariate associations with burnout dimensions, it was not retained in the final multivariate model, possibly reflecting shared variance with somatic symptom burden (*r* = 0.78, *p* < 0.001).

The model intercept was statistically significant (*B* = −4.964; *p* = 0.025), indicating a low estimated baseline log-odds of burnout when all predictors are at zero.

### 3.6 Model performance and predictive accuracy

The performance of the burnout exploratory association model demonstrated excellent discriminative ability. The confusion matrix (Table 5) revealed an overall accuracy of 94.7%, with a sensitivity of 93.9% and a precision of 94.5%. The model accurately classified the majority of residents with burnout, with a very low rate of false-positive and false-negative classifications.

**Table 5:** Confusion matrix of the statistical model for burnout prediction.

Actual burnout status	Predicted burnout	
Observed Burnout	NO	YES
NO (No-burnout)	165 (True Negative, TN)	8 (False Positive, FP)
YES (Burnout)	9 (False Negative, FN)	138 (True Positive, TP)

Values represent the number of correctly and incorrectly classified cases. “YES” indicates the presence of burnout as defined by the Maslach Burnout Inventory–Human Services Survey (MBI-HSS). **Model performance:** Accuracy = 94.7%; Sensitivity = 93.9%; Specificity = 95.4%; Precision = 94.5%.

**Figure 1:**
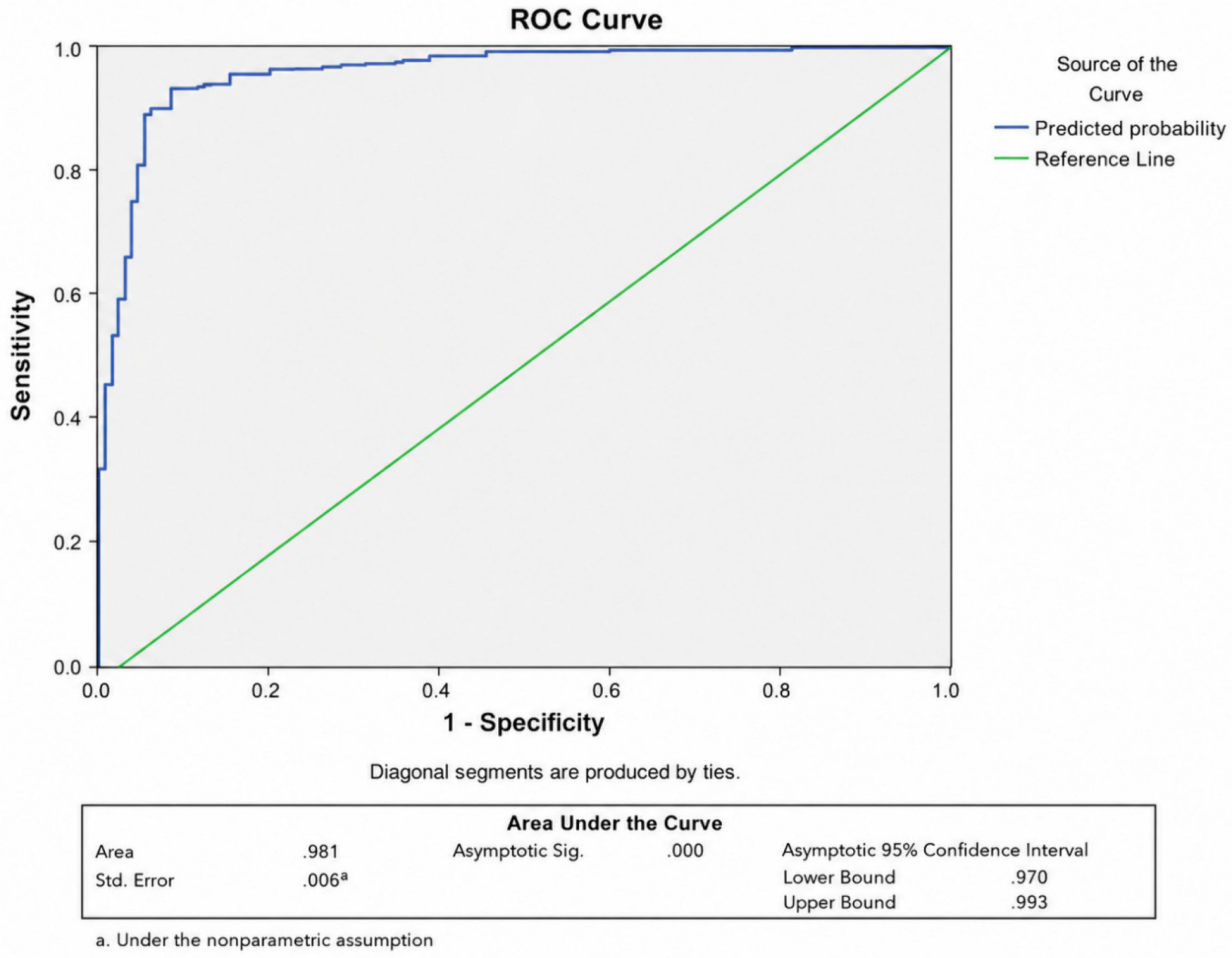
Receiver operating characteristic (ROC) curve of the logistic regression model. Note. Classification performance was calculated using a probability threshold of 0.50. The ROC curve was generated empirically using predicted probabilities derived from the final logistic regression model. The 95% confidence interval for the AUC was estimated using the DeLong non-parametric method.

These results were further supported by Receiver Operating Characteristic (ROC) curve analysis (Figure 1). The area under the curve (AUC) was 0.981 (95% CI: 0.970–0.993; *p* < 0.001), indicating an almost perfect ability to distinguish between burnout and non-burnout cases. Together, These findings indicate strong discriminative ability within the study sample; however, in the absence of validation procedures, the model should be interpreted cautiously as an exploratory analytical tool rather than a definitive predictive instrument.

Nevertheless, despite the excellent discriminative performance, external validation in independent samples is warranted to confirm the generalizability and stability of the model.

## 4 Discussion

The primary objective of this study aimed to determine the prevalence of burnout among Tunisian medical residents and to identify key associated factors. The findings revealed a high prevalence of burnout (45.9%), with mean scores indicating severe emotional exhaustion (34.62), depersonalization (19.06), and markedly reduced personal accomplishment (20.98). These results are consistent with the growing global concern regarding physician well-being, particularly among medical residents who are exposed to intense occupational demands.

Our findings align with previous studies reporting high levels of burnout among medical residents in different regions. Studies conducted in Brazil and Nigeria similarly documented severe burnout among residents, highlighting the universality of this phenomenon across diverse healthcare systems (49, 50). Furthermore, a recent meta-analysis among radiology residents reported that approximately half of participants experienced at least one dimension of burnout at a moderate to high level, reinforcing the widespread nature of this syndrome in postgraduate medical training (27). In line with existing literature, burnout prevalence appeared higher among residents in surgical and emergency-oriented specialties compared with those in medical specialties, likely reflecting differences in workload intensity and emotional burden.

The presence of strong correlations among variables such as insomnia severity and somatic symptoms reflects underlying psychosomatic interrelationships. While multicollinearity may influence coefficient stability, it does not invalidate the identification of associated factors in exploratory regression models.

### 4.1 The interplay of occupational strain, sleep architecture, and somatic manifestations

Our results support the critical role of night work and on-call burden as major contributors to burnout. Night shifts and extended working hours directly impair cognitive and physical functioning and progressively exhaust residents' adaptive resources, while also delaying physiological recovery processes. Previous research has demonstrated that fixed night-shift work is associated with an increased risk of sleep disturbances and mental health problems, underscoring the deleterious impact of circadian misalignment (51). Disruption of circadian rhythms has been shown to affect hormonal secretion patterns (52) and to increase the risk of cardiovascular disease (53) providing plausible biological pathways linking night work to burnout.

Moreover, the strong association observed between somatic symptoms and burnout dimensions lends support to the psychosomatic exploratory model of burnout. This model posits that chronic occupational stress is embodied through physiological dysregulation. Consistent with our findings, Hammarström et al. (29) reported a strong correlation between somatic complaints and psychological exhaustion, suggesting that physical symptoms may represent a somatic expression of prolonged emotional strain. Taken together, these results point toward a pathogenic cycle in which excessive workload and sleep disruption precipitate somatic symptoms, which in turn exacerbate emotional exhaustion and contribute to the progression of burnout.

### 4.2 Exercise as a multimodal protective intervention

One of the most important contributions of this study is the identification of regular physical activity as an associated protective factor against emotional exhaustion. The magnitude of this protective effect is noteworthy and aligns with a growing body of literature highlighting the role of exercise in mitigating occupational stress and burnout (8, 9). The beneficial effects of physical activity appear to be mediated through multiple pathways.

From a physiological perspective, regular exercise contributes to regulation of the hypothalamic–pituitary–adrenal (HPA) axis, reduces basal cortisol levels, and stimulates the release of endorphins and neurotrophic factors, thereby enhancing stress resilience (10, 11). Psychologically, physical activity may foster self-efficacy, emotional regulation, and cognitive detachment from work-related stressors (12). In addition, by improving sleep quality, exercise directly counteracts one of the most prominent risk factors for burnout identified in this and other studies (13).

Nevertheless, our findings should be interpreted within the context of ongoing debate in the literature. While several studies have reported a protective association between physical activity and burnout, others have failed to demonstrate a significant relationship (14, 15). These discrepancies may stem from methodological differences, including variations in the measurement of physical activity (self-report vs. objective assessment), exercise intensity and modality, and population-specific characteristics. The use of a validated questionnaire to assess habitual physical activity in our study strengthens the credibility of our findings and suggests that sustained engagement in physical activity, rather than occasional exercise, may be crucial for achieving protective effects (16).

### 4.3 Intrinsic motivation as a foundational resilience resource

Beyond occupational and lifestyle factors, our analysis highlighted a key psychological determinant of burnout resilience: motivational orientation. Residents who reported choosing their medical career based on intrinsic motivation were significantly less likely to experience burnout. This finding provides strong empirical support for Self-Determination Theory (SDT), which emphasizes the role of autonomous motivation in promoting psychological well-being (17).

According to SDT, behaviors driven by intrinsic interest and personal values are associated with greater persistence and well-being, whereas behaviors driven by external pressures or rewards are more likely to result in emotional exhaustion and ill-being (18).

Our results are consistent with evidence from other high-stress professions demonstrating that a sense of purpose and authentic engagement act as protective buffers against burnout (19). Importantly, these findings suggest that vulnerability to burnout may emerge early in the professional trajectory, during the process of career decision-making. Consequently, preventive strategies may benefit from being implemented at the level of medical education, with greater emphasis on career guidance that supports autonomy and personal alignment rather than external expectations or prestige (20).

### 4.4 Toward a predictive and preventative paradigm

The high discriminative capacity observed in the regression model suggests that the identified variables may be relevant markers of burnout risk. However, in line with methodological recommendations (e.g., TRIPOD Statement), the absence of calibration analysis and validation procedures precludes considering this model as a fully established predictive tool. The ability to accurately identify residents at high risk based on a limited set of occupational, psychological, and lifestyle factors represents a significant advance with direct translational relevance. Such predictive tools could be incorporated into routine occupational health or wellness assessments, enabling early, targeted interventions for vulnerable residents (21, 22).

By facilitating early identification and prevention, this approach may help reduce the progression to severe burnout, thereby safeguarding both physician well-being and the quality of patient care (23). Future research should focus on external validation of the model and on evaluating the effectiveness of intervention strategies informed by predictive risk profiling in real-world clinical training environments.

### 4.5 Limitations

Notwithstanding the advantages inherent in this multicenter investigation and the employment of established measurement tools, certain limitations ought to be considered. First, the cross-sectional design limits causal inferences regarding the identified associations between burnout and the occupational, psychological, and lifestyle-related factors. Longitudinal research is essential to ascertain temporal relationships and to evaluate whether modifications in workload, sleep quality, or physical activity engender significant reductions in burnout over an extended period. Second, all variables were evaluated through self-reported questionnaires, a method that may introduce reporting and recall biases, especially concerning sensitive behaviors like substance use or psychological distress. The reliance on self-reported history to exclude participants with diagnosed psychological disorders may have introduced misclassification bias, as undiagnosed or unreported conditions could not be objectively verified. While validated scales were utilized, self-report instruments might not provide a complete representation of objective workload intensity, sleep architecture, or physical activity profiles. Third, the study's participant pool comprised solely Tunisian medical residents within a limited age bracket, potentially restricting the applicability of the results to other healthcare systems, cultural environments, or phases of medical education. Moreover, unmeasured contextual variables, including institutional backing, supervisory effectiveness, or individual personality characteristics, could have affected burnout risk, yet were not incorporated into the current analysis. Despite the predictive model's strong internal performance, external validation remains pending. Subsequent research should assess the model's stability and utility across independent cohorts and varied clinical environments prior to its routine application. Furthermore, the number of on-call shifts per month was analyzed as a continuous predictor without formal testing of the linearity-in-the-logit assumption, which may affect the precision of the estimated effect size and should be considered when interpreting the associated odds ratio.

From a methodological standpoint, predictive modeling in clinical and epidemiological research requires rigorous development and validation processes, including calibration assessment, internal validation, and external validation across independent samples. Accordingly, the regression analysis presented here should be interpreted as exploratory, aimed at identifying key associated factors and generating hypotheses for future research, rather than establishing a clinically applicable prediction model.

### 4.6 Practical implications

The study results present several significant practical implications for both residency training programs and healthcare institutions. The robust correlation observed between on-call workload and burnout highlights the necessity of organizational interventions designed to optimize duty rosters, curtail excessive night shifts, and guarantee sufficient recuperation periods for residents. The identification of physical activity as a protective factor suggests that structured wellness initiatives should be actively integrated into residency programs. Providing access to exercise facilities, protected time for physical activity, and institutional support for healthy lifestyle behaviors may represent feasible and low-cost strategies to mitigate emotional exhaustion. Further, the protective role of intrinsic career motivation highlights the importance of early career guidance and mentorship. Medical education programs are encouraged to foster autonomy-supportive environments that encourage residents to align professional pathways with personal values and interests, thereby enhancing psychological resilience. Ultimately, the model's discriminative capacity suggests its potential relevance as a preliminary risk-stratification tool within occupational health services, pending external validation before any clinical application. The early identification of residents exhibiting a high risk of burnout could facilitate the implementation of targeted preventive measures, thereby supporting physician well-being and contributing to the enduring quality and sustainability of healthcare provision.

## 5 Conclusions

Burnout among Tunisian medical residents is a complex phenomenon influenced by occupational stress, sleep disturbances, somatic symptomatology, and motivational factors. This study identifies key associated factors, showing that intrinsic career motivation and regular physical activity may reduce burnout risk, whereas excessive on-call workload may intensify emotional exhaustion and depersonalization. The regression model highlights key factors associated with increased burnout risk and may provide a useful preliminary framework for future predictive model development, pending rigorous validation. These findings support the implementation of integrated, multilevel strategies encompassing workload optimization, health promotion, and psychological support to safeguard physician well-being and sustain healthcare system performance. Future research should prioritize external model validation and the evaluation of targeted preventive interventions within residency training settings.

## Data Availability

The raw data supporting the conclusions of this article will be made available by the authors, without undue reservation.
